# The causes and management of nonunion of femoral subtrochanteric shortening osteotomy in a THA patient: a case report

**DOI:** 10.1186/s12891-019-2612-2

**Published:** 2019-05-10

**Authors:** Song Gong, Weihua Xu, Ruoyu Wang, Shaokai Liu, Lizhi Han, Guo Chen, Bo Wang

**Affiliations:** 1 Department of Orthopaedics, Union Hospital, Tongji Medical College, Huazhong University of Science and Technology, Wuhan 430022, China; 2 Department of Rehabilitation, Union Hospital, Tongji Medical College, Huazhong University of Science and Technology, Wuhan 430022, China

**Keywords:** Nonunion, Subtrochanteric transverse osteotomy, Crowe type IV DDH, THA, Internal fixation

## Abstract

**Background:**

Total hip arthroplasty (THA) is considerably difficult to perform in patients with Crowe type IV developmental dysplasia of the hip (DDH). Some Crowe type IV DDH patients require a femoral subtrochanteric shortening osteotomy to equalize the length of the lower extremities and decrease the difficulty of intraoperative reduction. Subtrochanteric transverse osteotomy has been proven to have superior clinical efficacy, but some cases of nonunion occur.

**Case presentation:**

We present the case of a 62-year-old male patient who underwent right THA with femoral subtrochanteric transverse osteotomy due to Crowe type IV DDH. Nonunion of the osteotomy occurred during the follow-up period. In July 2017, the patient underwent right THA and femoral subtrochanteric transverse osteotomy due to Crowe type IV DDH. In November 2017, a slight feeling of bone rubbing and slight pain in the hip were reported. The ends of the osteotomy had rotated and united poorly. However, the patient requested to undergo continued observation. In December 2017, the patient reported an obvious sensation of bone rubbing and aggravated hip pain. The ends of the osteotomy had rotated and continued to exhibit nonunion. On December 26, 2017, the patient was treated with plate and screw internal fixation with bone morphogenetic protein (BMP) following our suggestion. In August 2018, the ends of the osteotomy had united after internal fixation was applied.

**Conclusions:**

THA with femoral subtrochanteric transverse osteotomy exhibits good efficacy for the treatment of patients with Crowe type IV DDH. However, postoperative nonunion occurs in a small number of cases. The causes of nonunion should be analysed, and effective measures should be taken to prevent this situation. Plate and screw internal fixation with BMP is an effective treatment for nonunion of the ends of an osteotomy.

## Background

Total hip arthroplasty is recommended for patients with developmental dysplasia of the hip who remain disabled after receiving conservative treatment alone [1]. However, THA is a highly difficult surgery in patients with Crowe type IV DDH. Some Crowe type IV DDH patients undergoing THA require a femoral shortening osteotomy to equalize the length of the lower extremities and decrease the difficulty of intraoperative reduction and the incidence of complications [2–4]. The femoral subtrochanteric transverse osteotomy is one of the most common forms of osteotomy [5, 6]. Although a subtrochanteric transverse osteotomy has significant efficacy for the treatment of Crowe type IV DDH patients [4, 7], some complications still occur, including intraoperative osteotomy fracture, neurovascular injury, postoperative osteotomy nonunion, and hip dislocation, [6, 8]. These complications seriously affect the postoperative rehabilitation of patients. Our study reports the case of a patient with type IV DDH who underwent THA and experienced nonunion after transverse osteotomy. The causes, prevention and treatment of nonunion after transverse osteotomy are discussed based on literature reports.

## Case presentation

**Fig. 1:**
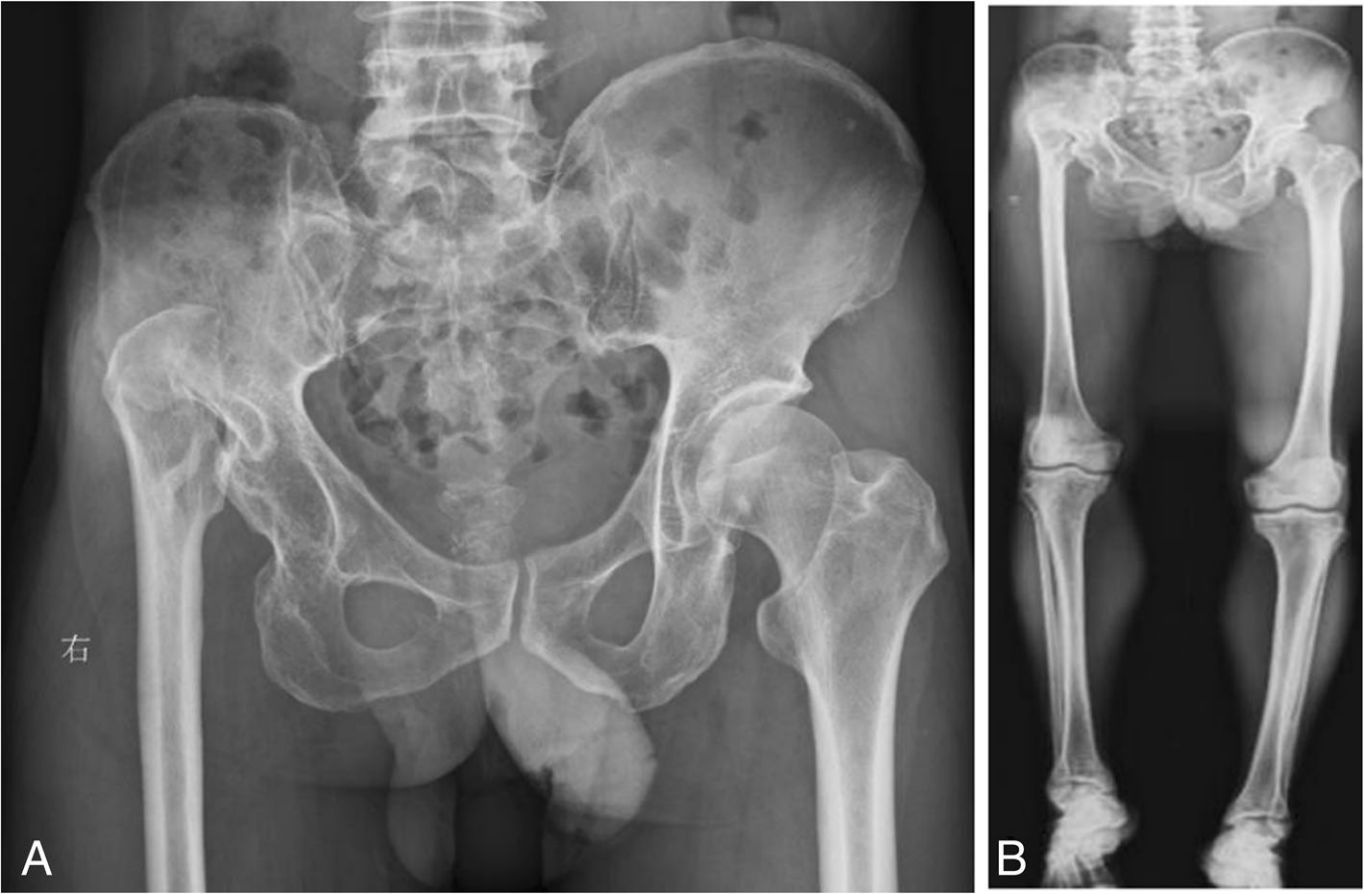
Radiographs of the pelvis and both lower extremities before the operation. (**a**) anteroposterior view of the pelvis, (**b**) anteroposterior view of both lower extremities

A 62-year-old male patient was admitted to the hospital due to a 50-year history of intermittent pain and limited activity of the right hip that had been aggravated for 1 month. A radiograph of the pelvis showed high dislocation of the right hip (Fig. 1), and the patient was diagnosed with right Crowe type IV DDH. The patient’s visual analogue scale (VAS) score was 8, and his Harris score was 21. A physical examination showed that the right lower extremity was shortened by 5.3 cm, and the patient had a limp, local tenderness of the right hip, and aggravated pain upon internal and external rotation of the hip. The degrees of right hip joint motion were as follows: flexion, 90°; outreach, 12°; adduction, 14°; internal rotation 10°; and external rotation, 5°. The patient reported no other disease history.

**Fig. 2:**
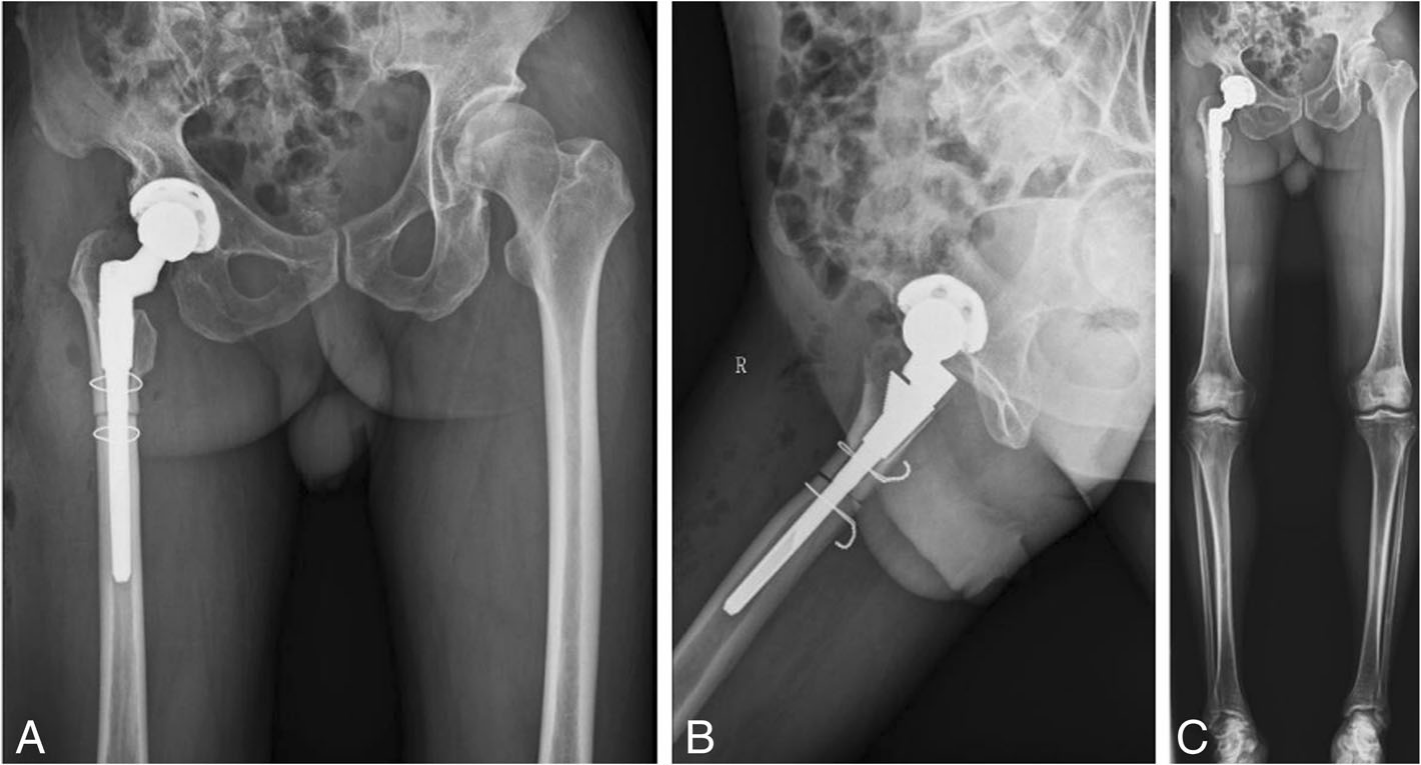
Radiographs of both lower extremities and the right hip after the operation. (**a**) anteroposterior view of the right hip, (**b**) lateral view of the right hip, (**c**) anteroposterior view of both lower extremities

**Fig. 3:**
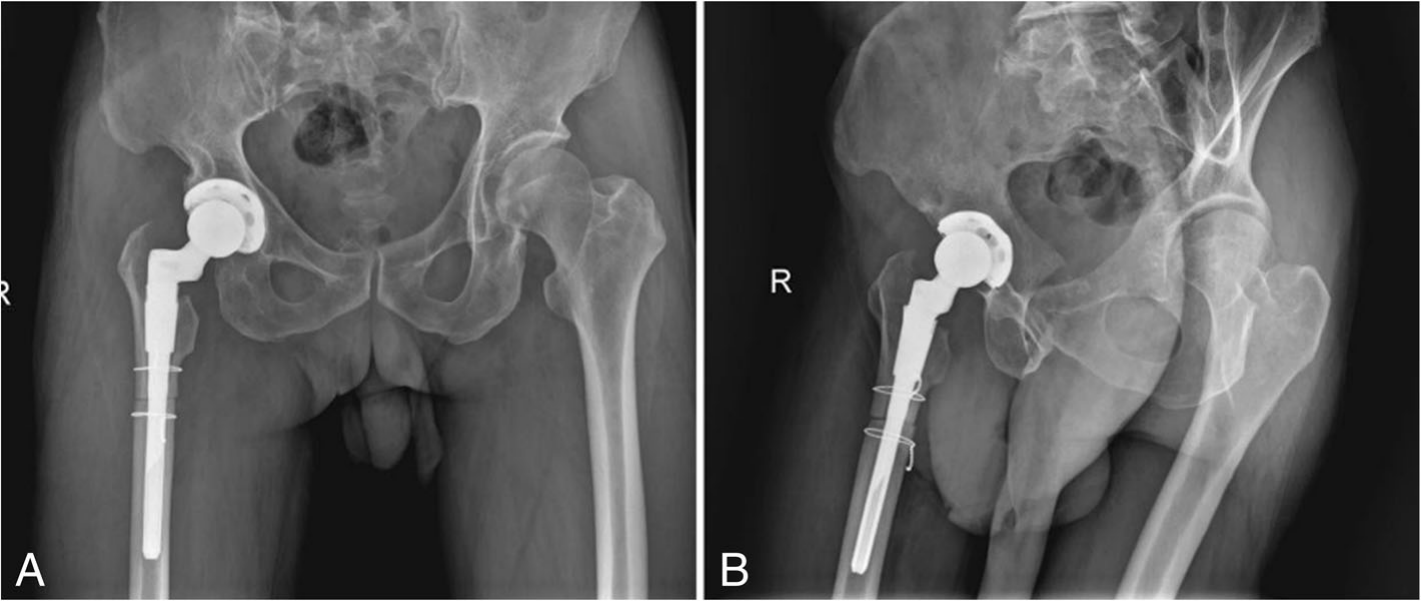
Radiographs of the right hip at 40 days after the operation. (**a**) anteroposterior view of the right hip, (**b**) lateral view of the right hip

**Fig. 4:**
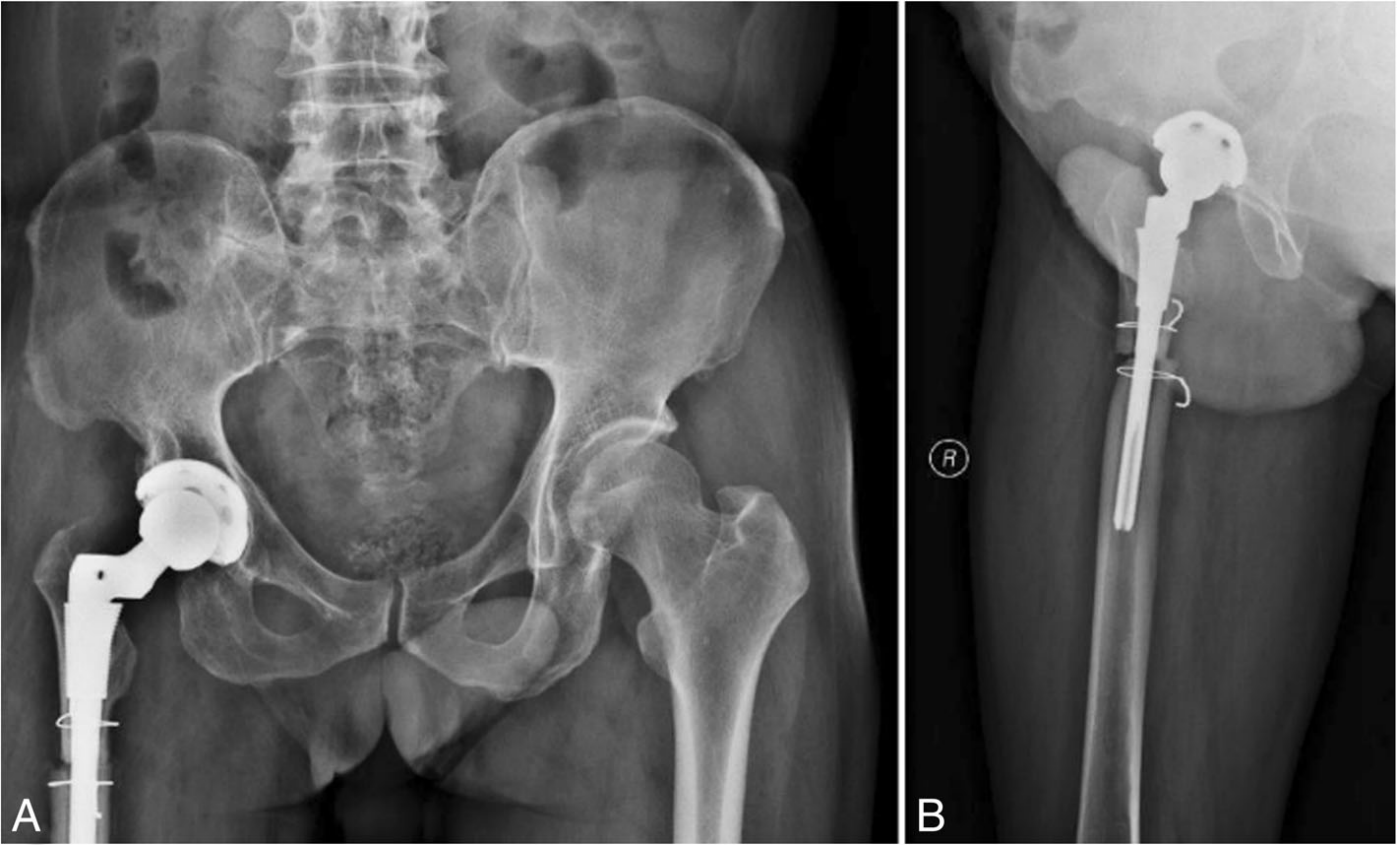
Radiographs of the right hip at three and a half months after the operation. (**a**) anteroposterior view of the right hip, (**b**) lateral view of the right hip

**Fig. 5:**
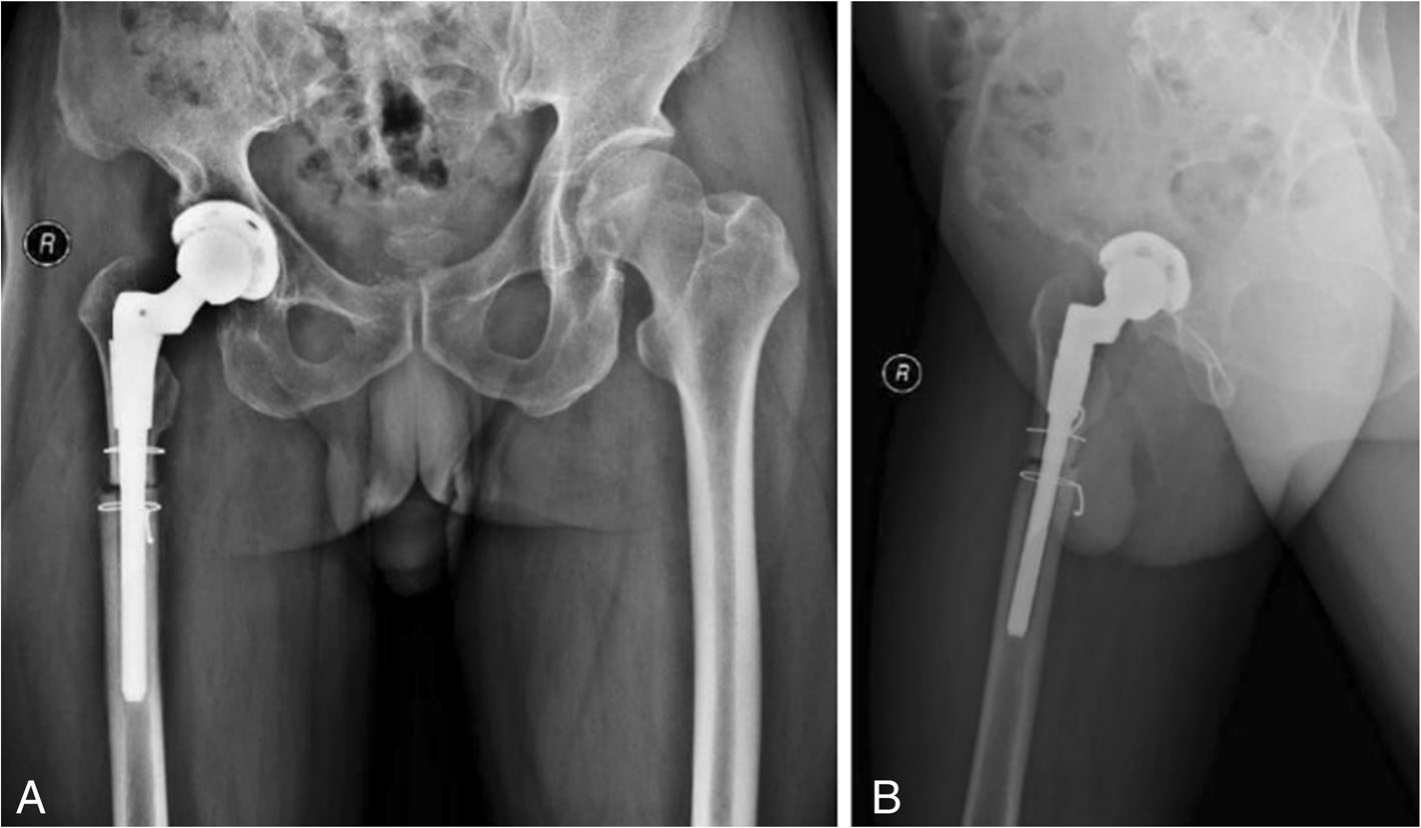
Radiographs of the right hip at 5 months after the operation. (**a**) anteroposterior view of the right hip, (**b**) lateral view of the right hip

The patient was treated with right THA and femoral subtrochanteric shortening transverse osteotomy. The osteotomy was located 1.6 cm below the lesser trochanter, the length of the osteotomy was 2.7 cm, and steel wires were attached at both ends of the osteotomy to prevent fracture. The intraoperative characteristics of the Johnson company S-ROM prosthesis are as follows: bio-type, 44 mm acetabular cup, 28 mm polyethylene liner, 28 mm ceramic head, and standard shank. The patient was treated postoperatively with antibiotics, analgesics, anticoagulants, and gastroprotectants. A radiograph of both lower extremities and the right hip showed satisfactory positioning of the prosthesis after the operation (Fig. 2). One day after surgery, the patient could walk in the ward with the help of a walker. One week after surgery, the patient could walk freely but limped because of an evidently tilted pelvis. The patient’s VAS score was 3, and his Harris score was 60. At 40 days after surgery, the patient could put on his socks by himself, and the limp and pelvic tilt were significantly corrected after following the training regimen of the rehabilitation plan. The patient was instructed to practice squatting to facilitate using the toilet. The two ends of osteotomy were in good contact with each other (Fig. 3). The patient’s VAS score was 2, and his Harris score was 79. Three and a half months after the operation, the patient reported a slight sensation of bone rubbing and mild pain in the operated hip. The patient’s VAS score was 3, and his Harris score was 70. The ends of the osteotomy had rotated and united poorly (Fig. 4). We recommended that the patient undergo hospitalization to receive internal fixation, but the patient requested to continue observation. Five months after the operation, the patient had obvious sensations of bone rubbing, and his right hip pain was significantly worse. The patient’s VAS score was 5, and his Harris score was 52. The ends of the osteotomy had rotated and exhibited nonunion (Fig. 5). The patient had obvious symptoms of pain, and hospitalization for surgery was recommended.

**Fig. 6:**
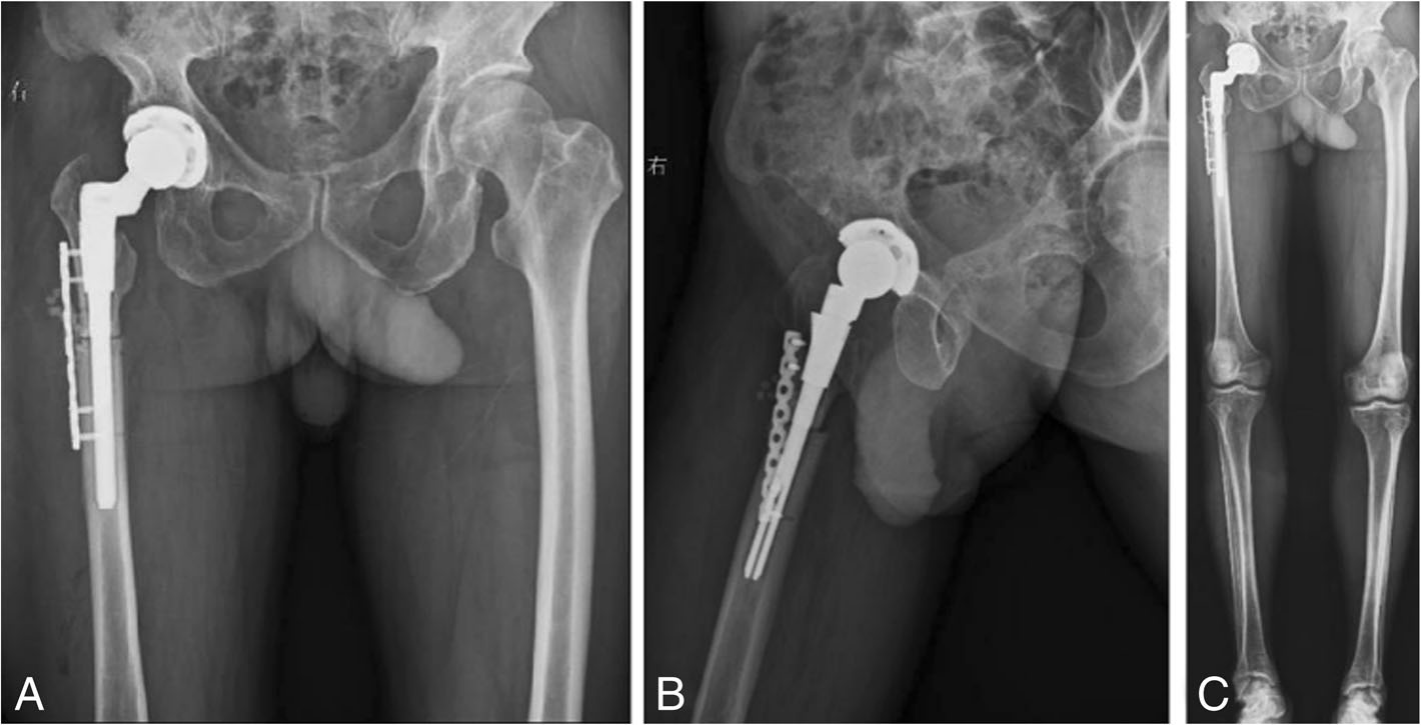
Radiographs of the right hip and both lower extremities after internal fixation was applied. (**a**) anteroposterior view of the right hip, (**b**) lateral view of the right hip, (**c**) anteroposterior view of both lower extremities

**Fig. 7:**
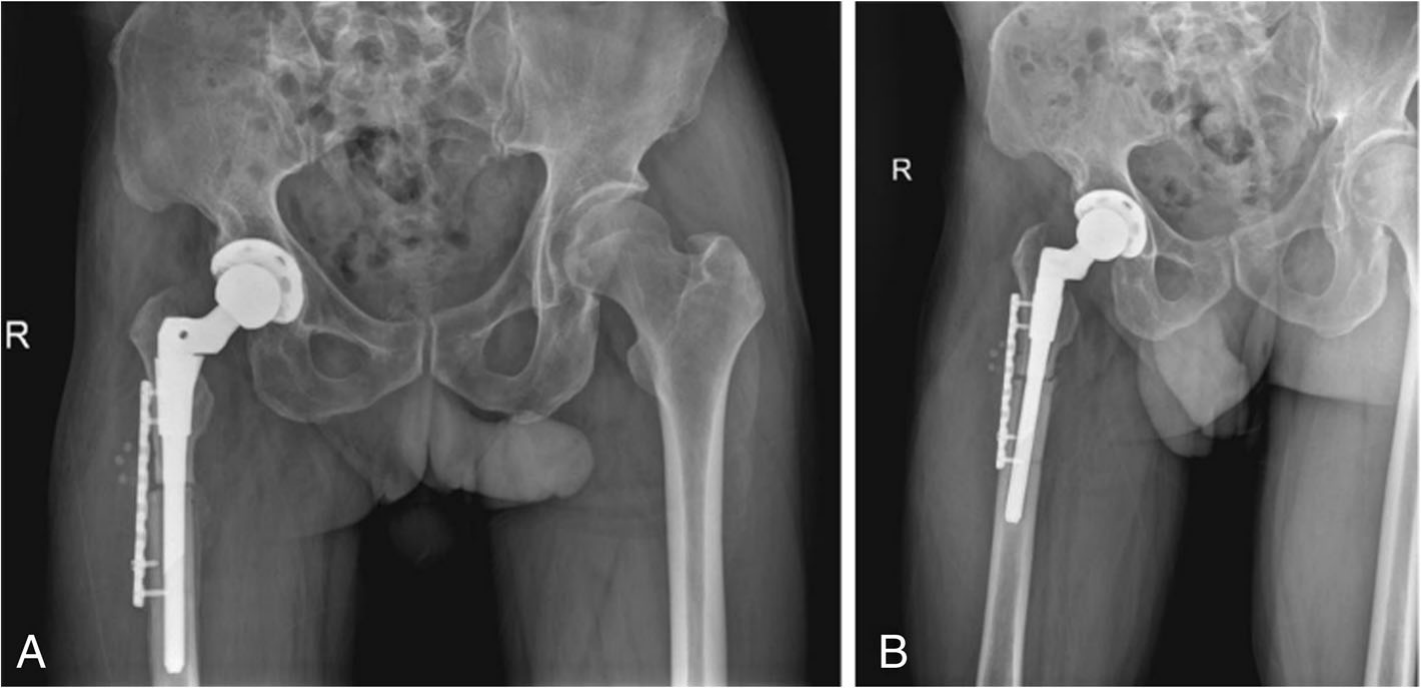
Radiographs of the right hip at 1 month after internal fixation was applied. (**a**) anteroposterior view of the right hip, (**b**) lateral view of the right hip

**Fig. 8:**
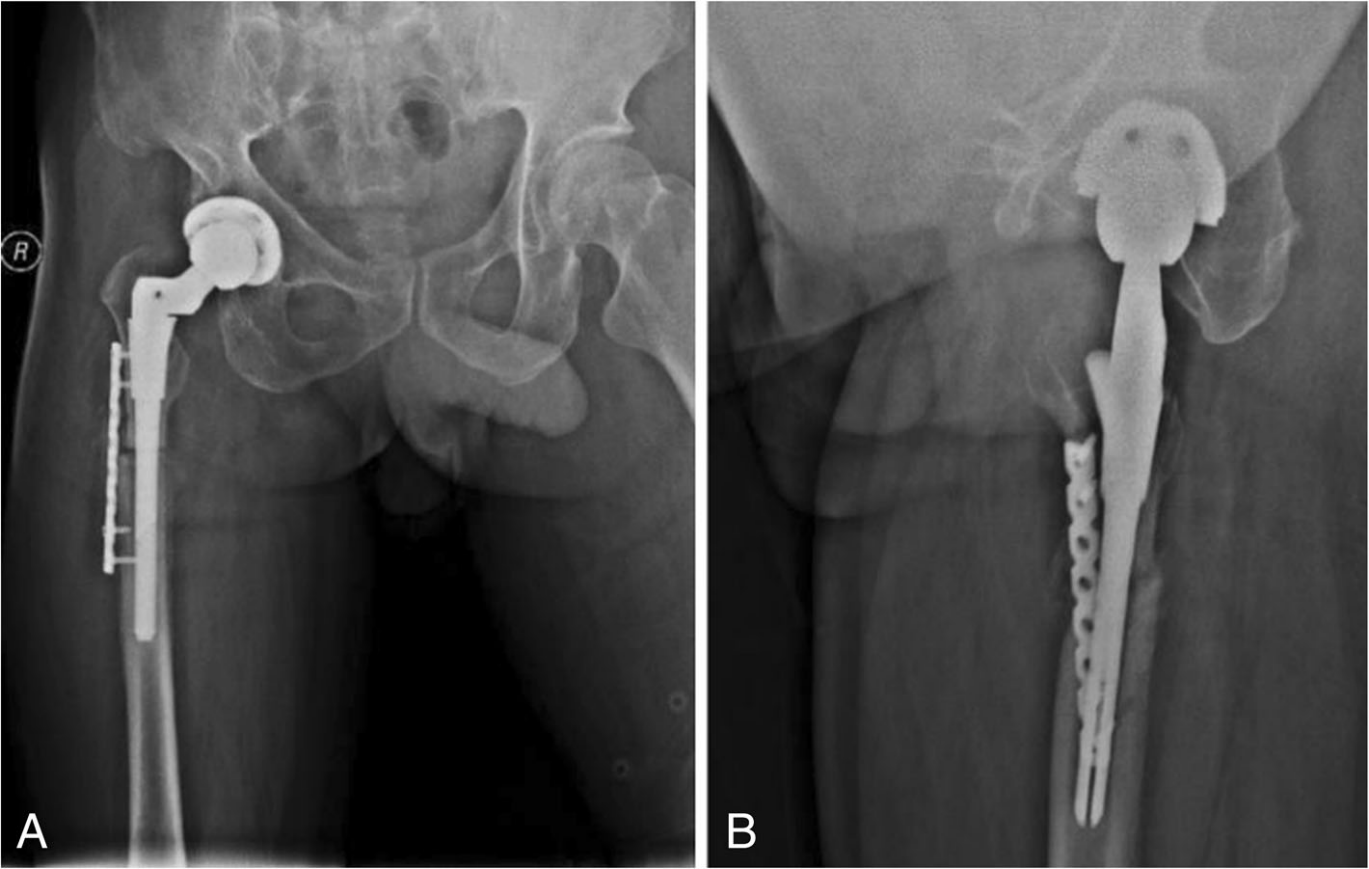
Radiographs of the right hip at two and a half months after internal fixation was applied. (**a**) anteroposterior view of the right hip, (**b**) lateral view of the right hip

**Fig. 9:**
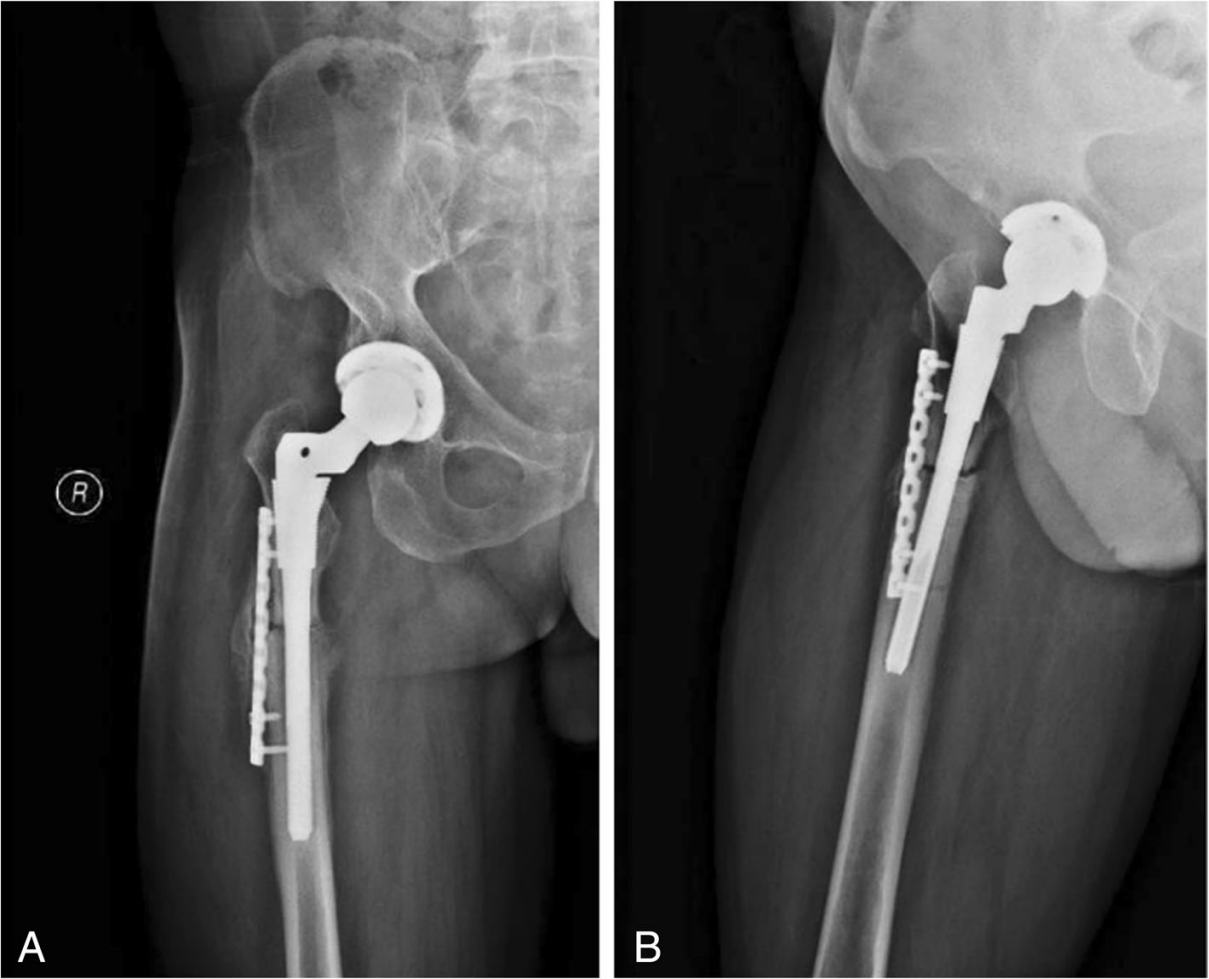
Radiographs of the right hip at 5 months after internal fixation was applied. (**a**) anteroposterior view of the right hip, (**b**) lateral view of the right hip

**Fig. 10:**
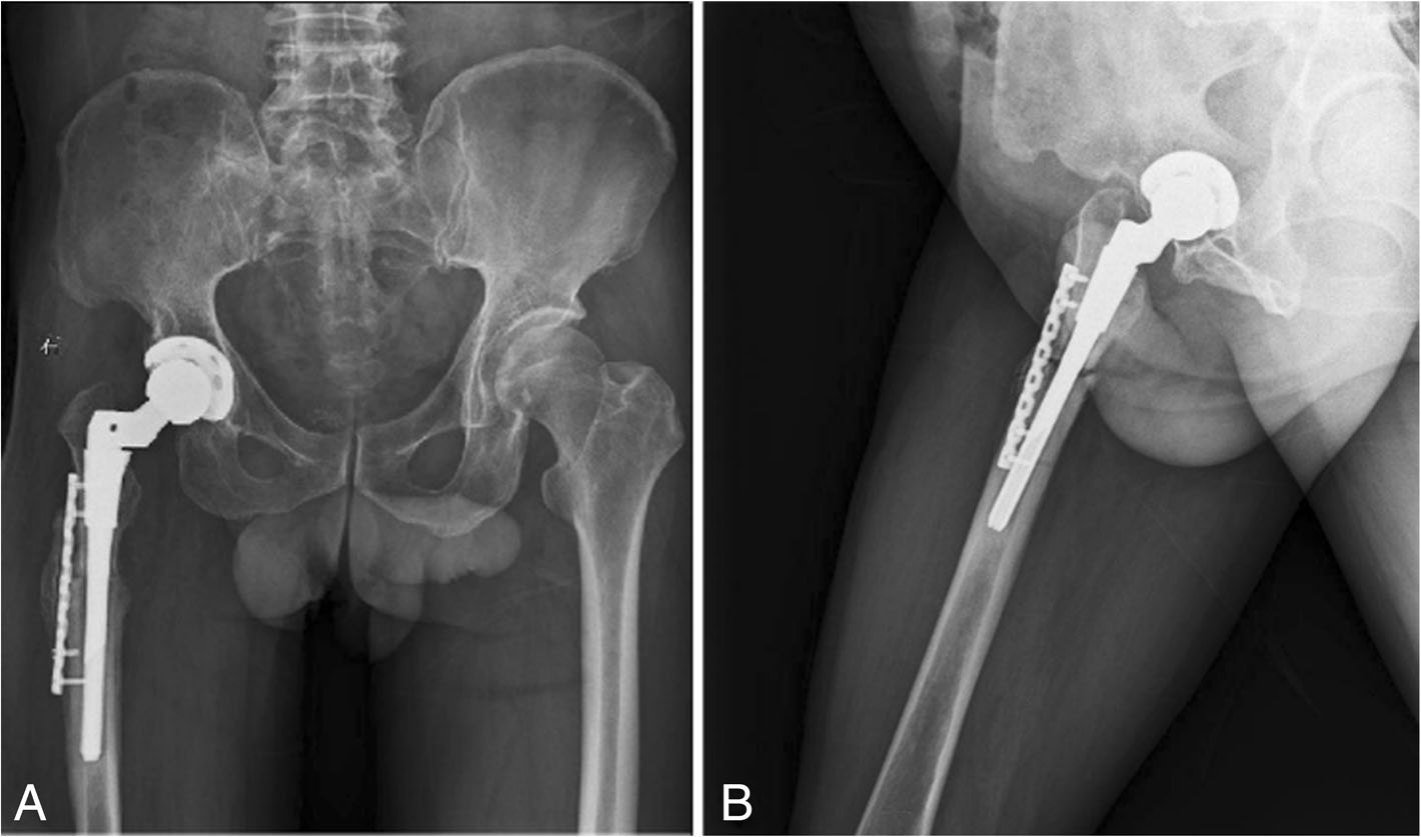
Radiographs of the right hip at 8 months after internal fixation was applied. (**a**) anteroposterior view of the right hip, (**b**) lateral view of the right hip

The patient was diagnosed with nonunion of the osteotomy and was again admitted for plate and screw internal fixation with bone morphogenetic protein (BMP). The patient was treated postoperatively with antibiotics, analgesics, anticoagulants, and gastroprotectants. Three days after surgery, the patient reported a significant reduction in pain, and the sensation of bone rubbing was absent. The patient’s VAS score was 3, and his Harris score was 60. A radiograph of the right hip and both lower extremities showed that the plate and screw was well fixed (Fig. 6). One month after internal fixation was applied, the patient reported that his situation was good. The patient’s VAS score was 2, and his Harris score was 78. The ends of the osteotomy had not rotated and had begun to exhibit union (Fig. 7). Two and a half months after internal fixation was applied, the patient’s VAS score was 2, and his Harris score was 85. The ends of the osteotomy were firmly fixed, and the union was progressing satisfactorily (Fig. 8). Five months after internal fixation was applied, the patient had only occasional slight pain that did not affect his life. The patient’s VAS score was 1, and his Harris score was 87. Bone scabs had formed at the ends of the osteotomy (Fig. 9). Eight months after internal fixation was applied, the patient reported no problems with normal activities. His VAS score was 0, and his Harris score was 90. The ends of the osteotomy had united (Fig. 10).

## Discussion

High dislocation of the femoral head and soft tissue contracture around the hip in patients with Crowe type IV DDH make intraoperative hip joint reduction difficult [8]. Femoral osteotomy can be performed to decrease the difficulty of intraoperative reduction and the incidence rate of complications caused by forced reduction in these patients [2–4, 9, 10]. Although multiple osteotomy methods can be used for patients with Crowe type IV DDH, the femoral subtrochanteric shortening transverse osteotomy is one of the most common techniques [5, 6].

Femoral subtrochanteric transverse osteotomy has been clinically shown to have satisfactory efficacy. However, some cases of nonunion still occur. According to literature reports, the nonunion rate of transverse osteotomy is 1.3~20% (Table 1) [2–4, 6, 11–17]. The possible reasons for the occurrence of nonunion, as in our case, are as follows. First, excessive periosteal stripping may have reduced or interrupted the blood supply to the osteotomy site, resulting in nonunion of the osteotomy [2, 4, 8, 13]. Second, the high local temperature of the saw may have affected the union of the osteotomy site [4, 15]. Third, the process of reaming can injure the endosteum and cause local necrosis of the bone marrow cavity, which may loosen an originally stable prosthesis and affect union [7, 12, 13]. Fourth, incomplete matching of the round stem and the femoral medullary elliptical cavity may result in rotation and affect union. Fifth, soft tissue embedded in the osteotomy ends may affect union. Sixth, premature load or inappropriate activity may lead to nonunion.

**Table 1:** Summary of the literature on osteotomy nonunion

Study	Total	Nonunion	Percentage
Akiyama et al. [11]	15	3	20%
Charity et al. [12]	18	1	5.6%
Wang et al. [13]	56	2	3.6%
Sofu et al. [14]	73	4	5.5%
Wang et al. [4]	76	1	1.3%
Mu et al. [15]	71	2	2.8%
Ollivier et al. [3]	28	2	7.1%
Krych et al. [2]	28	2	7.1%
Masonis et al. [16]	24	2	8.3%
Park et al. [6]	24	3	12.5%
Yalcin et al. [17]	44	5	8.8%

Effective measures should be taken to avoid nonunion in these difficult cases. First, a large and sufficiently long distal femoral stem should be selected to prevent rotation, which can result in nonunion [6, 18]. Second, the selected femoral prosthesis could be relatively small for patients undergoing surgery of the minor proximal medullary cavity. The distal femoral stem does not have a sufficient ability to prevent rotation. Plate and screw internal fixation is a good choice to increase the stability of the osteotomy ends and promote union [19, 20]. Third, the ends of the osteotomy need to be as smooth as possible to increase contact [4, 15]. Fourth, periosteal stripping should be reduced during the osteotomy procedure [4]. Fifth, application of cold saline flushing is beneficial to reduce damage to bone tissue during osteotomy.The S-ROM femoral prosthesis is applied using the following procedure. The proximal cuff, which has a rough outer surface, is tightly attached to the interior part of the proximal femur. The inner part of the proximal cuff is strongly attached to the tapered femoral stem. The longitudinal streak of the distal portion of the femoral stem is tightly pressed against the medullary cavity. This configuration prevents the S-ROM femoral prosthesis from rotating [7, 18]. The prosthesis may rotate because the distal femoral stem is too small or the implantation force of the femoral prosthesis is not sufficient. In our case, the S-ROM femoral prosthesis may not have prevented rotation, which impacted the union of the osteotomy ends. Therefore, the medullary cavity of the femur should be fully reamed, and an S-ROM femoral prosthesis that matches the distal femur should be used.

The most common methods of performing subtrochanteric osteotomy include transverse, step-cut, or double chevron techniques [5, 15, 16]. The two most prominent advantages of subtrochanteric transverse osteotomy are simple operation and a freely adjustable anteversion angle. The disadvantage of this osteotomy is that the incidence of nonunion is relatively high compared to that of other of osteotomy types. Subtrochanteric step-cut and double chevron osteotomies have a relatively low incidence of nonunion, but these two osteotomy methods have higher technical requirements. The major disadvantage of these methods is that adjustment of anteversion is difficult if the osteotomy fails. In our case, rotational instability of the transverse osteotomy led to nonunion.

The following improvements will be implemented for similar cases in the future. The size and type of the femoral medullary cavity should be carefully evaluated preoperatively. The femoral medullary cavity should be sufficiently reamed intraoperatively. A long and sufficiently large femoral prosthesis should be selected to prevent rotation. We currently use a cortical bone plate with a steel cable or plate and screw internal fixation to increase the stability of the osteotomy ends. We will continue to perform and utilize the advantages of subtrochanteric transverse osteotomy while taking effective measures to avoid its associated complications.

## Conclusions

THA with femoral subtrochanteric transverse osteotomy can provide good clinical efficacy and result in a relatively high rate of union in Crowe type IV DDH patients. However, some patients still experience nonunion. The causes of nonunion should be carefully analysed, and measures should be actively taken to prevent its occurrence. Plate and screw internal fixation with BMP is an effective treatment choice if nonunion occurs.

## Abbreviations

BMP: Bone morphogenetic protein
DDH: Developmental dysplasia of the hip
THA: Total hip arthroplasty
VAS: Visual analogue scale
